# Hypnotizability and Time Reproduction

**DOI:** 10.1007/s12311-026-02005-2

**Published:** 2026-04-28

**Authors:** Žan Zelič, Valentina Puccini, Enrica L. Santarcangelo

**Affiliations:** 1 Department of Physics, University of Trento, Trento, Italy; 2 Department of Translational Research and New Technologies in Medicine and Surgery, University of Pisa, Pisa, Italy

**Keywords:** Time perception, Cerebellum, Insular cortex, Hypnosis

## Abstract

Hypnotizability is a psychophysiological trait associated with structural variation in brain regions implicated in time perception, such as the cerebellum and the insula. This study investigated time reproduction across different interval durations in individuals with varying levels of hypnotizability. Forty healthy, right-handed participants of both sexes were assessed using the Stanford Hypnotic Susceptibility Scale: Form A and completed a time reproduction task consisting of visually presented intervals of 1, 3, 6, and 8 s (10 trials per duration). Relative proportional error (RE) and absolute proportional error (AE) were analyzed using mixed-effects models estimating the interaction between hypnotizability and interval duration through polynomial contrasts, with random intercepts and duration slopes for participants. Heart rate, sex, trait anxiety, and absorption were included as covariates. RE showed only a significant main effect of interval duration, with shorter intervals overestimated and longer intervals underestimated. AE showed a significant interaction between hypnotizability and the linear component of interval duration, indicating that higher hypnotizability was associated with a steeper decrease in AE as interval duration increased. The relatively lower precision at shorter intervals is consistent with attenuated sensory processing, whereas the relatively higher precision at longer intervals may reflect better functional coupling between executive and salience networks previously observed in highly hypnotizable individuals. The reduced precision at shorter intervals may be more consistent with cerebellar than insular involvement. Differences in time perception may be linked to reported associations between hypnotizability and an altered sense of agency and body ownership.

**Supplementary Information:**

The online version contains supplementary material available at 10.1007/s12311-026-02005-2.

## Introduction

Hypnotizability is a stable trait [1, 2] that predicts the proneness to experience suggested alterations in physiology, emotion, cognition, and behavior following the hypnotic induction [3], but also responsiveness to non-hypnotic suggestions and psychophysiological functioning in the ordinary state of consciousness [4]. It is measured by standardized scales, whose scores are commonly used to categorize individuals as high (highs), medium (mediums), or low (lows) hypnotizable. However, due to variability in classification criteria across studies and information loss associated with the categorization approach, recent research favors treating hypnotizability as a continuous dimension [5].

Neuroimaging studies show morpho-functional brain differences between highs and lows, such as the highs’ higher functional connectivity between the anterior cingulate and the dorsolateral prefrontal cortex [6], and reduced gray matter volume (GMV) in the insula [7, 8] and left cerebellar lobules IV-VI [8]. Smaller insular GMV may be associated with highs’ less accurate perception of interoceptive signals [9–11] and lower amplitude of the heartbeat-evoked cortical potential [12, 13]. Smaller cerebellar GMV may be responsible for highs’ less precise sensorimotor control and altered sensorimotor learning patterns [14, 15], and for their higher resting excitability of the right motor cortex [16], likely due to reduced cerebellar inhibition [17, 18]. Both the insula and cerebellum serve predictive and comparator functions, forming internal models of motor actions and bodily states, including their temporal sequences [19]. This motivates the hypothesis that hypnotizability-related variation in these structures may also extend to time experience. For instance, highs perceive visual and auditory stimuli as simultaneous over a wider range of onset asynchronies than lows [20], suggesting reduced temporal precision.

### Time Perception

Timing is a key component of many physiological and psychological functions [21]. Recent behavioral evidence suggests a common factor underlying time perception across different time intervals [22], while meta-analytic neuroimaging evidence [23, 24] indicates that timing engages a distributed set of partially overlapping networks involving the supplementary motor area, insula, basal ganglia, cerebellum, and fronto-parietal association cortices, with their relative contribution depending on the specific task and interval duration [19]. Perception of short time intervals (below 1.2 s) may be based more on sensory processing, whereas estimating longer intervals (above 1.2 s) may require more complex cognitive processes [25].

The cerebellum is primarily engaged in processing sub-second timing intervals and fine-grained coordination of events across the sensorimotor, cognitive, and emotional domains [19, 26, 27]. Behavioral, neuroimaging, and lesion studies demonstrate its involvement in the production, reproduction, and discrimination of time intervals, rhythmic finger tapping, and sensorimotor synchronization across the millisecond range [19, 28, 29]. However, left cerebellar lesions also impair the timing of suprasecond intervals [30], likely due to the cerebellum’s interactions with the prefrontal cortex [31]. Indeed, cerebellar dentate nucleus activity monitors timing in both sub-second and suprasecond intervals [32]. The cerebellar lobule VI, characterized by reduced GMV in highs [8], has been identified as a core node in timing [28, 33].

The insula integrates bodily signals and exteroceptive information into higher-order representations of body states [34, 35] and is consistently involved in time perception across intervals of various lengths [19]. Insular damage is associated with less precise temporal estimation [36]. In time reproduction tasks, insular activation increases with the interval length [37, 38], and the connectivity of the posterior insula, whose GMV is lower in highs than in lows [7], is positively associated with reproduction accuracy [39]. Moreover, the reproduced duration is associated with changes in the amplitude of the late component of the heartbeat-evoked cortical potential [40], which has been proposed to reflect elaborative processing of cardiac information [41] and differs between highs and lows [12, 13].

### Aim of the Study

Given the reduced insular and cerebellar GMV, lower interoceptive accuracy, and lower sensorimotor precision observed in highs compared to lows [42], hypnotizability-related differences in time perception can be hypothesized. Thus, the primary aim of the study was to examine the relative (RE) and absolute proportional error (AE) in the reproduction of time intervals of different durations as a function of hypnotizability. Moreover, we investigated the possible role of heart rate, anxiety, and absorption, which are reported to influence time perception [43, 44]. The findings may provide further insight into the physiology of hypnotizability and the clinical relevance of its assessment for the development of new interventions [42].

## Materials and Methods

The study was conducted in accordance with the Declaration of Helsinki and approved by the Bioethics Committee of the University of Pisa (N. 8/2025, February 21). Written informed consent to participate in the study and for the publication of anonymized results was obtained from all participants before enrollment.

### Participants

One hundred and twelve students at the University of Pisa had been tested for hypnotizability by the Italian version of the Stanford Hypnotic Susceptibility Scale: Form A (SHSS: A [45]), for absorption by MODTAS [46], which is a modified version of the Tellegen Absorption Scale (TAS [47]), and for trait anxiety (STAI-Y2 [48, 49]) in the preceding 6 months. Among them, 46 consecutive volunteers agreed to participate in the experimental session. Inclusion criteria were age between 18 and 35 years and normal or corrected-to-normal vision. Exclusion criteria were the history of medical, neurological, and psychiatric disorders, sleep and attention disorders, and ongoing psychoactive therapies. Due to the previously observed association between handedness and time perception [50], we also excluded non-right-handed participants using a short Italian version of the Edinburgh Handedness Inventory [51, 52]. After excluding six volunteers (three for ongoing pharmacological therapies, one for a PTSD diagnosis, and two for left-handedness), the final sample consisted of 40 participants (24 females, 16 males; mean *±* SD: age = 24.05 *±* 2.86; SHSS score = 5.33 *±* 3.74). The sample included 16 lows (SHSS: A score *≤* 4 out of 12), 10 mediums (score 5–7), and 14 highs (score *≥* 8).

### Experimental Procedure

**Fig. 1:**
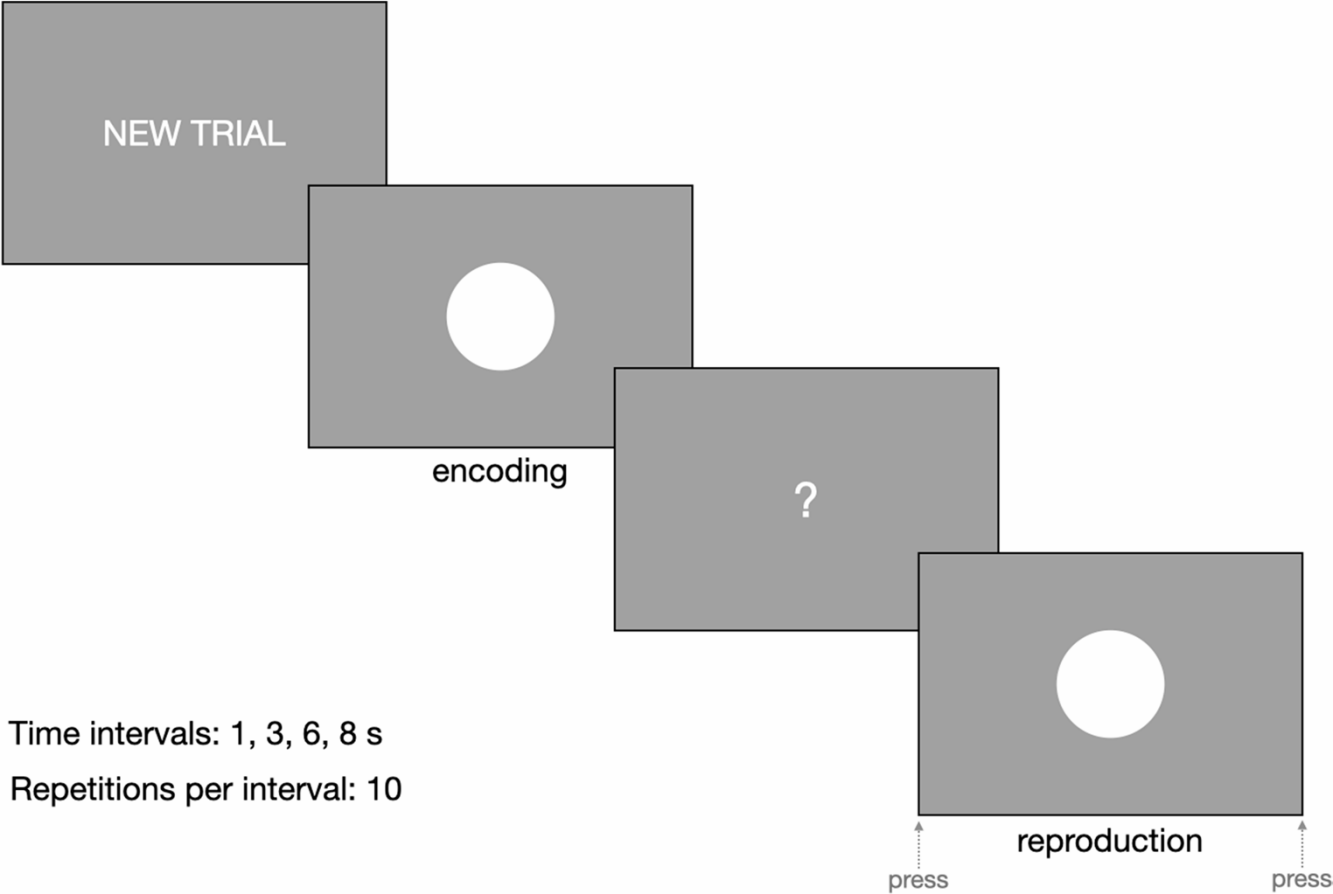
Design of the time reproduction task

Upon arrival at the laboratory, the participants were invited to sit in a sound- and light-attenuated room in front of a computer screen and were fitted with ECG electrodes. Next, they read the written instructions for the time reproduction task and received all necessary explanations to ensure they understood the task (Fig. 1). Before starting the experiment, participants completed a training trial to familiarize themselves with the task and were allowed to ask additional questions.

### Variables

#### Hypnotizability (SHSS: A)

Hypnotizability was measured using the Stanford Hypnotic Susceptibility Scale: Form A (SHSS: A [45]), which consists of a standardized hypnotic induction with fixation and a set of 12 suggestions. The experimenter observes the behavioral responses to suggestions and scores them on a dichotomous scale. According to the standard classification, individuals scoring *≤* 4 are classified as lows, 5–7 as mediums, and *≥* 8 as highs. The scale’s internal consistency was good (Cronbach’s ɑ = 0.87).

#### Absorption (MODTAS)

We measured the tendency to become deeply involved in ongoing experiences using a modified version (MODTAS; [46]) of the Tellegen Absorption Scale, whose score correlates with hypnotizability (TAS [47]). MODTAS comprises 34 items, forming five subscales (synaesthesia, altered state of consciousness, aesthetic involvement, imaginative involvement, extrasensory perception) and a single higher-order factor (Cronbach’s ɑ = 0.93). In contrast to the original dichotomous response format, each item is evaluated on a 5-point Likert scale ranging from 0 (never) to 4 (very often).

#### Trait Anxiety (STAI-Y2)

Since anxiety may modulate time perception [53], we administered the trait form of the State-Trait Anxiety Inventory (STAI-Y2 [48, 49]), which consists of 20 items scored on a four-point scale ranging from 1 (not at all) to 4 (very much). The scale’s internal consistency was excellent (Cronbach’s ɑ = 0.90). 

#### Time Reproduction Error

To assess time perception, we used a time reproduction task, which is one of the main methods in timing research and is widely used [54]. Compared with other established paradigms, such as temporal bisection, time reproduction gives trial level errors, is less dependent on anchor learning and decision criteria, and can easily accommodate different target durations in one session. We selected the version of the reproduction task in which participants press a key both at start and at the end of the reproduction interval, as this version has shown higher overall precision than others [55].

The task was implemented in PsychoPy as a standard visual time reproduction paradigm (Fig. 1) [55]. Each trial consisted of an encoding phase (experiencing the time interval) and a reproduction phase (reproducing the previously observed time interval by pressing a key). During the encoding phase, a white circle appeared at the center of the screen on a uniform gray background for a given duration. Participants were asked to carefully observe its duration and to reproduce it as accurately as possible in the following step. Then, a question mark appeared, marking the start of the reproduction phase. Participants had to press the keyboard spacebar to make the circle reappear. When they judged that the interval matched the previously observed interval duration, they had to press the spacebar again to make the circle disappear. The duration intervals were 1, 3, 6, and 8 s, each repeated 10 times (for a total of 40 trials), presented in a randomized order. Before each trial, a screen displaying “NEW TRIAL” was introduced to reduce possible confusion. Short breaks between the presentation of the different screens were jittered (sampling from 1, 1.25, 1.5, 1.75, 2 s) to reduce temporal predictability.

#### Heart Rate

To estimate possible effects of heart rate (HR) on time reproduction, we monitored the ECG at the standard D1 derivation using disposable Ag/AgCl electrodes (FIAB, Vicchio, Firenze, Italy) during the time reproduction task. The ECG signal was acquired using a Psylab system (Contact Precision Instruments, Boston, USA). The Pan-Tompkins algorithm [56] was used to identify R peaks, followed by visual inspection and manual correction of misrecognized peaks. The data were also screened for possible extrasystoles and bundle branch blocks (none of which were found in any participant).

### Statistical Analysis

Preliminary analyses examined correlations between hypnotizability, heart rate, anxiety, and absorption. Independent-samples *t*-tests were used to examine sex differences in all variables. Following previous research [55], we analyzed the relative proportional error (RE) and the absolute proportional error (AE), computed as:

$$\mathrm{RE} = (\text{reproduced duration - actual duration})/\text{actual duration}$$

$$\mathrm{AE} = |\text{reproduced duration - actual duration}| / \text{actual duration}$$

RE and AE were analyzed using mixed-effects models with fixed effects for hypnotizability (SHSS: A score), interval duration (1, 3, 6, 8 s), and their interaction. Random intercepts and random slopes for duration were included for participants. Duration was modeled as an ordered factor using a priori orthogonal polynomial contrasts (linear, quadratic, and cubic components [57]). Sex, heart rate, trait anxiety, and absorption were examined as separate covariates. All continuous predictors were z-transformed before analysis. RE was analyzed using a linear mixed-effects model estimated by maximum likelihood, with fixed effects evaluated using Satterthwaite-approximated *t*-tests. Given the strongly right-skewed distribution of AE, we applied a generalized linear mixed-effects model with a Gamma distribution and log link, with fixed effects evaluated using Wald *z*-tests. Model assumptions for RE were assessed using residual versus fitted plots and Q-Q plots of residuals. For AE, model fit was evaluated using GLMM residual diagnostics (including dispersion and residual simulations). A variance inflation factor (VIF) below 5 was considered indicative of the absence of multicollinearity problems. All analyses were performed in *R*, using the *psych*, *lme4*,* glmmTMB*,* lmerTest*, and *emmeans* packages. The significance level was set at ɑ < 0.05.

## Results

SHSS: A scores positively correlated with STAI-Y2 (*r* = .53, *p* < .001) and MODTAS total scores (*r =* .55, *p* < .001). No significant sex differences were observed in SHSS: A, STAI-Y2, MODTAS, and HR. Descriptive statistics for all variables are presented in Table S1 (Suppl. El. Mat.).

### Relative Error

**Fig. 2:**
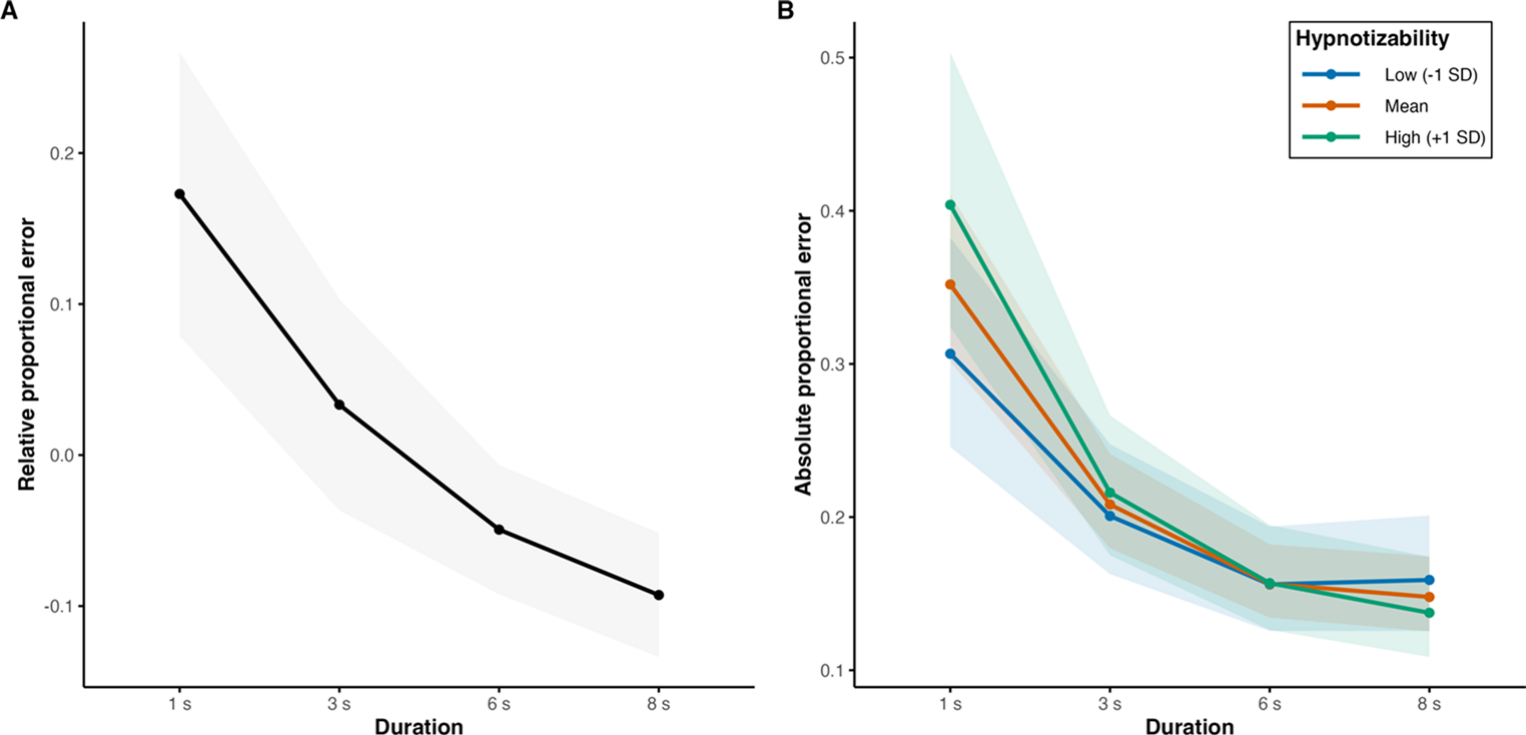
Relative (RE) and Absolute Error (AE). RE (**A**) varies with the interval duration, AE (**B**) varies with hypnotizability and interval duration. The ribbons represent 95% confidence intervals

A linear mixed-effects model predicting RE using a priori polynomial contrasts for duration showed a significant effect of interval duration, with significant linear (*b* = -0.20, *SE* = 0.04, *t*(40.00) = -5.15, *p* < .001) and quadratic components (*b* = 0.05, *SE* = 0.01, *t*(1520.01) = 3.49, *p* < .001) and no support for the cubic component (*b* = -0.02, *SE* = 0.01, *t*(1520.01) = -1.47, *p* = .143). Descriptively, shorter intervals were overestimated, and longer intervals were underestimated (Fig. 2A). There was no evidence for a main hypnotizability effect (*b* = 0.04, *SE* = 0.02, *t*(40.00) = 1.65, *p* = .107) or for an interaction between hypnotizability and interval duration (linear: *b* = -0.02, *SE* = 0.04, t(40.00) = -0.65, *p* = .522; quadratic: *b* = -0.02, *SE* = 0.01, *t*(1520.01) = -1.60, *p* = .111; cubic: *b* = 0.01, *SE* = 0.01, *t*(1520.01) = 0.87, *p* = .384). Including sex, anxiety, absorption, and HR as separate covariates did not change the results.

### Absolute Error

A Gamma generalized linear mixed-effects model (with a log link) predicting AE showed a significant effect of interval duration, with significant linear (*b* = -0.64, *SE* = 0.07, *z* = -9.85, *p* < .001) and quadratic components (*b* = 0.23, *SE* = 0.04, *z* = 5.28, *p* < .001). There was no evidence for a main hypnotizability effect (*b* = 0.03, *SE* = 0.07, *z* = 0.40, *p* = .690). However, we observed a significant interaction between hypnotizability and the linear component of duration (linear: *b* = -0.15, *SE* = 0.07, *z* = -2.20, *p* = .028; quadratic: *b* = 0.01, *SE* = 0.05, *z* = 0.29, *p* = .773; cubic: *b* = -0.04, *SE* = 0.04, *z* = -0.81, *p* = .416). Descriptively, AE was relatively higher at shorter intervals and lower at longer intervals with increasing hypnotizability (Fig. 2B**).** Including sex, HR, absorption, and anxiety as separate covariates did not modify the results.

## Discussion

The study aimed to assess whether hypnotizability, which is associated with insular and cerebellar GMV variations [7, 8] and related physiological and behavioral differences [42], also predicts performance on a visual time reproduction task. The results indicate that the precision of the interval reproduction (AE), but not the directional error in timing (RE), shows duration-dependent association with hypnotizability.

Our findings show that RE depends on interval duration independently of hypnotizability, with shorter intervals being overestimated and longer intervals being underestimated, as observed in the general population [58]. This is consistent with the central tendency effect [59], according to which, in uncertain conditions, temporal judgements tend to approach the estimated mean value of the presented intervals. Our findings also align with previous evidence indicating no association between hypnotizability and directional time estimation in the ordinary state of consciousness [60]. Nonetheless, after neutral hypnosis, the interval durations have been judged as shorter in retrospective timing paradigms [61, 62] and as longer in prospective paradigms [60]. Moreover, during hypnosis, no significant association between hypnotizability and time perception has been reported [60–62]. Attentional load and expectancies related to hypnosis may account for differences between hypnotized and non-hypnotized individuals [63, 64], whereas relaxation does not appear to explain hypnotic time distortion [65].

In contrast to RE, AE showed an interaction between hypnotizability and interval duration. Higher hypnotizability was associated with a steeper decrease in AE with increasing duration, indicating relatively higher AE at shorter intervals and relatively lower AE at longer intervals. These hypnotizability-related differences in AE across intervals may reflect the differential contributions of sensory processing, which is more relevant for shorter intervals, and cognitive processing, which is more relevant for longer intervals [25]. Indeed, highs exhibit attenuated somatosensory [66] and interoceptive [12, 13] processing compared to lows, which may be related to their smaller cerebellar and insular GMV [7, 8], and may have impaired their performance at short intervals. In contrast, highs exhibit stronger functional connectivity between executive and salience networks [6], which may have improved their performance at longer intervals. The present findings do not allow us to disentangle the relative contributions of the insula and cerebellum to temporal precision, as both participate in a broader predictive allostatic network [67] and have been implicated in timing across short and long intervals [19, 30–32]. Nevertheless, the observed pattern may be more consistent with a role of the cerebellum, which is more relevant to the shortest intervals than the insula, whose activity increases with increasing interval duration [37, 38].

In the present study, the effects observed for RE and AE were unchanged when controlling for anxiety, sex, and absorption. Previous findings suggest that the effects of these variables on time perception depend strongly on task context and methodological characteristics. Anxiety has been associated with time underestimation when threat was experimentally induced [44]. Sex differences in duration judgements are generally small and depend on the experimental paradigm [68]. Similarly, the effect of absorption varies across task contexts [43]. In the present study, the prospective paradigm may have contributed to the absence of sex differences, as they are more pronounced in retrospective paradigms [43]. The emotionally neutral nature of the task may have reduced the role of anxiety, whereas the monotonous and repetitive trials may have reduced task engagement. The observed absence of heart rate contribution to time perception is also consistent with evidence that subjective arousal, rather than heart rate itself, influences time perception [69], as the task employed in the present study lacked emotional valence.

Larger AE in shorter intervals may at least partially account for the highs’ attenuated sense of agency [70, 71] and body ownership [72]. The sense of agency depends on predictions regarding the timing and consequences of actions, and both the insula [73, 74] and the cerebellum have been implicated in agency through their predictive functions [75, 76]. Indeed, highs experience more pronounced feelings of involuntariness and effortlessness in action both during hypnosis and in the ordinary state of consciousness [70]. Moreover, in the ordinary state of consciousness, highs exhibit delayed awareness of intention and noisier action-time estimates [77], as well as reduced metacognition of agency when lags are introduced between actions and their consequences [71]. Highs’ lower precision in reproducing shorter intervals could induce weaker coupling of intentions, actions, and perceived outcomes, resulting in an altered experience of agency. The rubber hand illusion also demonstrates that the general population experiences an altered sense of body ownership depending on the temporal association between multisensory signals [78], and that highs are more prone than lows to body ownership alteration [72]. The present findings may have broader clinical relevance, as disturbances in agency and body ownership are implicated in various neuropsychiatric conditions, such as dissociative disorders, posttraumatic stress disorder, and functional neurological disorder [79–81], which are more frequent in highs [82–85].

A limitation of the study is that the different methods used to measure time perception may yield different results [54, 55]. Similarly, timing performance may depend on the sensory modality of the presented stimuli [54, 86]. Moreover, the number of trials performed in the present study was relatively modest, which may have reduced sensitivity to small effects. However, a substantial increase in trial number could lead to fatigue and attention diversion. Also, state anxiety was not measured immediately before the time reproduction task, and therefore only trait anxiety could be considered as a covariate. Another limitation is that reaction times were not measured, which would have allowed us to rule out a motor bias that may have influenced the estimation of shorter intervals more than the longer ones. However, in the ordinary state of consciousness, shorter reaction times have been observed in highs compared to lows [87, 88]. Thus, it is unlikely that the highs’relatively larger error in reproducing shorter intervals was due to reaction times. Finally, to clarify the association of GMV variations in the cerebellum and insula with time reproduction errors, future research should include behavioral and physiological measures of cerebellar and insular functioning. The inclusion of sub-second intervals could also help to further disentangle their contribution to hypnotizability-related differences in time perception.

## Summary

Time perception engages some parts of the neural circuits that show hypnotizability-related structural and functional differences. In the present study, time reproduction RE was not associated with hypnotizability, whereas AE showed that higher hypnotizability was associated with a steeper decrease in error with increasing interval duration. Theoretically, this finding may be supported by hypnotizability-related variations in insular and cerebellar GMV, and with the reported differences in coupling between executive and salience networks. Although the present findings do not allow us to disentangle insular from cerebellar contributions to time reproduction, the differences across time intervals suggest that the hypnotizability-related cerebellar variation may have been more influential than the insular variation. The hypnotizability-related time reproduction error may also partially explain the differences in agency and body ownership between highs and lows.

## Electronic Supplementary Material

Below is the link to the electronic supplementary material.

Supplementary Material 1

## Data Availability

The data are available upon request.
