# Supplementary material for: Hypnotizability and Time Reproduction
Source: Cerebellum. 2026 Apr 28;25(3):63. doi: 10.1007/s12311-026-02005-2 (PMC13124866; doi:10.1007/s12311-026-02005-2)
Supplement: Supplementary file 1 — Supplementary Material 1 [file 12311_2026_2005_MOESM1_ESM.docx]

**Supplementary Electronic Material**

**Table S1.** *Descriptive statistics of all measured variables and sex differences*

|  |  | **All** (*N* = 40) | |  | **Females** (*N* = 24) | |  | **Males** (*N* = 16) | |  | **sex difference *t*-test** | |
| --- | --- | --- | --- | --- | --- | --- | --- | --- | --- | --- | --- | --- |
|  |  | ***M*** | ***SD*** |  | ***M*** | ***SD*** |  | ***M*** | ***SD*** |  | ***t*** | ***p*** |
| **SHSS: A** |  | 5.32 | 3.74 |  | 5.83 | 3.91 |  | 4.56 | 3.46 |  | 1.08 | .288 |
| **MODTAS** |  | 57.05 | 21.57 |  | 55.88 | 23.52 |  | 58.81 | 18.86 |  | 0.44 | .665 |
| **STAI-Y2** |  | 49.02 | 10.54 |  | 49.42 | 11.02 |  | 48.44 | 10.09 |  | 0.29 | .774 |
| **HR** |  | 78.81 | 12.29 |  | 80.97 | 11.65 |  | 75.56 | 12.89 |  | 1.35 | .187 |
| **RE** | **1s** | .17 | .34 |  | .13 | .29 |  | .23 | .41 |  | 0.81 | .426 |
|  | **3s** | .03 | .19 |  | .02 | .21 |  | .05 | .17 |  | 0.45 | .654 |
|  | **6s** | -.05 | .14 |  | -.05 | .15 |  | -.05 | .14 |  | 1.35 | .894 |
|  | **8s** | -.09 | .10 |  | -.10 | .11 |  | -.09 | .09 |  | 0.33 | .743 |
| **AE** | **1s** | .39 | .22 |  | .36 | .17 |  | .43 | .28 |  | 0.84 | .411 |
|  | **3s** | .22 | .11 |  | .23 | .11 |  | .20 | .10 |  | 0.96 | .345 |
|  | **6s** | .17 | .08 |  | .17 | .08 |  | .17 | .09 |  | 0.06 | .950 |
|  | **8s** | .16 | .08 |  | .17 | .08 |  | .14 | .07 |  | 1.39 | .174 |
